# Genome Sequence of a Clinical *Staphylococcus aureus* Strain from a Prosthetic Joint Infection

**DOI:** 10.1128/genomeA.00198-16

**Published:** 2016-04-07

**Authors:** Claire Marquès, Christine Franceschi, Valérie Collin, Frédéric Laurent, Sonia Chatellier, Christiane Forestier

**Affiliations:** a bioMérieux SA, La-Balme-les-Grottes, France; b Laboratoire Microorganismes: Génome et Environnement, UMR CNRS 6023, Université d’Auvergne, Clermont Ferrand, France; c Department of Clinical Microbiology, Hospices Civils de Lyon, Lyon, France; d INSERM U1111, International Center for Research in Infectiology, University of Lyon 1, CNRS UMR 5308, Lyon, France

## Abstract

Here, we report the genome sequence of *Staphylococcus aureus* LYO-S2, an isolate with sequence type (ST) 45 that was isolated in 2001 from a prosthetic joint infection.

## GENOME ANNOUNCEMENT

*Staphylococcus aureus* is a Gram-positive ubiquitous bacterial species that is considered to be an important pathogen that causes hospital- and community-acquired infective diseases. The resulting infections are difficult to treat because of frequent resistance to commonly used antibiotics and the ability of the strains to form biofilms (1, 2).

Here, we report the genome sequence of *S. aureus* strain LYO-S2, isolated in 2001 from a patient suffering from a prosthetic joint infection after total knee arthroplasty in the public hospital of Lyon, France. The strain belongs to multilocus sequence type 45 (3) and is methicillin-susceptible.

The genomic DNA from LYO-S2 was sequenced by next-generation sequencing using an Illumina HiSeq 2500 instrument with 125-bp paired-end reads. The genome was assembled by SOAPdenovo2 v1.03 software (4), resulting in 50,307,694 reads with an average length of 125 pb, which assembled into 24 scaffolds ranging from 214 bp to 1,275,288 bp, and resulted in a total genomic length of 2,681,263 nt. The G+C content was 32.71%. The open reading frames (ORFs) were obtained by the software MyRast and were annotated by the Figfams database (5). Genome annotation resulted in 2,538 annoted genes, including 278 hypothetical proteins and 55 tRNAs.

The LYO-S2 genome contains the *ica* operon (for the synthesis of extracellular poly-N-acetyl-glucosamine), accessory gene regulator (*agrB* and *D*), elastin-binding adhesins (*ebpS)*, fibronectin (*fnbA* and *B*), and collagen (*cna*), and multiple genes encoding staphylococcal enterotoxins, exotoxins, and superantigens.

Genes involved in resistance to antibiotics such as methicillin-resistance proteins (*fmtA* and *B*, *hmrA*, and *femC*), multidrug-resistance protein B, drug-resistance transporter (*emrB/qacA* subfamily), teicoplanin resistance-associated membrane proteins (*tcaA*, *B*, and *R*), quinolone-resistance protein (*norA*), and chloramphenicol-resistance protein were also found. Moreover, some genes coding for resistance to metals such as cobalt-, zinc-, cadmium-, and aluminum-resistance proteins were identified, as well as genes encoding toxic anion- and glyoxalase/bleomycin-resistance proteins.

### Nucleotide sequence accession numbers.

This whole-genome shotgun project has been deposited at DDBJ/EMBL/GenBank under the accession numbers FCOV01000001 to FCOV01000024. The study identification number is PRJEB12461.
